# A dual mechanism of action of AT-527 against SARS-CoV-2 polymerase

**DOI:** 10.1038/s41467-022-28113-1

**Published:** 2022-02-02

**Authors:** Ashleigh Shannon, Véronique Fattorini, Bhawna Sama, Barbara Selisko, Mikael Feracci, Camille Falcou, Pierre Gauffre, Priscila El Kazzi, Adrien Delpal, Etienne Decroly, Karine Alvarez, Cécilia Eydoux, Jean-Claude Guillemot, Adel Moussa, Steven S. Good, Paolo La Colla, Kai Lin, Jean-Pierre Sommadossi, Yingxiao Zhu, Xiaodong Yan, Hui Shi, François Ferron, Bruno Canard

**Affiliations:** 1 Architecture et Fonction des Macromolécules Biologiques, CNRS and Aix Marseille Université, UMR 7257, Polytech Case 925, 13009 Marseille, France; 2 Atea Pharmaceuticals, Inc., 125 Summer Street, Suite 1675, Boston, MA 02110 USA; 3 Università degli Studi di Cagliari, Monserrato, Italy; 4 Wuxi Biortus Biosciences Co. Ltd, 214437 Jiangyin, Jiangsu China; 5 European Virus Bioinformatics Center, Leutragraben 1, 07743 Jena, Germany

**Keywords:** Multienzyme complexes, Cryoelectron microscopy

## Abstract

The guanosine analog AT-527 represents a promising candidate against Severe Acute Respiratory Syndrome coronavirus type 2 (SARS-CoV-2). AT-527 recently entered phase III clinical trials for the treatment of COVID-19. Once in cells, AT-527 is converted into its triphosphate form, AT-9010, that presumably targets the viral RNA-dependent RNA polymerase (RdRp, nsp12), for incorporation into viral RNA. Here we report a 2.98 Å cryo-EM structure of the SARS-CoV-2 nsp12-nsp7-nsp8_2_-RNA complex, showing AT-9010 bound at three sites of nsp12. In the RdRp active-site, one AT-9010 is incorporated at the 3′ end of the RNA product strand. Its modified ribose group (2′-fluoro, 2′-methyl) prevents correct alignment of the incoming NTP, in this case a second AT-9010, causing immediate termination of RNA synthesis. The third AT-9010 is bound to the N-terminal domain of nsp12 - known as the NiRAN. In contrast to native NTPs, AT-9010 is in a flipped orientation in the active-site, with its guanine base unexpectedly occupying a previously unnoticed cavity. AT-9010 outcompetes all native nucleotides for NiRAN binding, inhibiting its nucleotidyltransferase activity. The dual mechanism of action of AT-527 at both RdRp and NiRAN active sites represents a promising research avenue against COVID-19.

## Abstract (web summary)

The drug AT-527 targets the SARS-CoV-2 replication machinery. Here the authors use Cryo-EM to show how AT-527 inhibits SARS-CoV-2 polymerase by acting as an immediate RNA chain terminator and stably binding in a NiRAN active-site pocket; impeding an essential nucleotide-transfer activity.

## Introduction

**Fig. 1:**
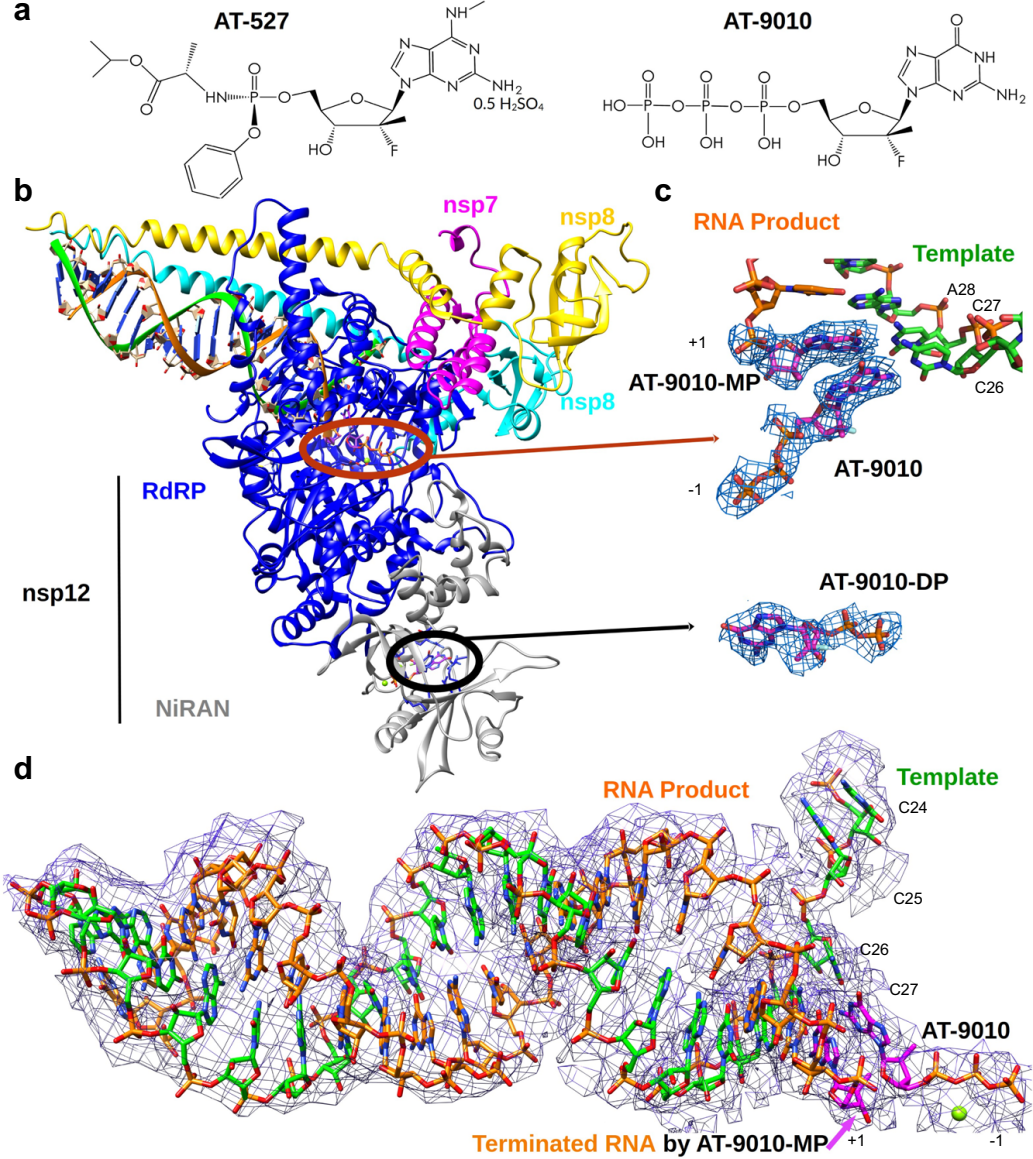
Cryo-EM structure of the RTC with bound RNA and AT-9010 molecules and corresponding cryo-EM map. **a** Structure of the guanine analog phosphoramidate prodrug AT-527 (left) and its active triphosphate form AT-9010 (right) following activation by cellular kinases. **b** Ribbon and stick representation of the cryo-EM structure of nsp7-(nsp8)_2_-nsp12:AT-9010-terminated-RNA:(AT-9010)_2_ complex. RNA, AT-9010 and protein shown with the following colors: template RNA, green; RNA product, orange; AT-9010, magenta; nsp7, pink; nsp8_1_ and nsp8_2_, yellow and cyan respectively; and nsp12 in gray and blue for NiRAN and RdRp domains respectively. **c** Brown and black ovals are enlarged for RdRp domain and NiRAN domains, respectively, showing experimental cryo-EM map around AT-9010, In the RdRp, one AT-9010 monophosphate (AT-9010-MP) is incorporated at (+1) position, with an incoming AT-9010 occupying the (−1) position. In the NiRAN domain, AT-9010 is bound it its diphosphate form (AT-9010-DP). **d** Experimental cryo-EM map for RNA (stick representation), showing incorporated AT-9010-MP (+1) and incoming AT-9010 (−1). Bases of the template RNA involved in the interaction with AT-9010 are numbered as C24–C27. All cryo-EM maps are represented at 3.5σ representative of the general map of the entire complex at 2.98 Å resolution.

The highly conserved replicative enzymes of positive-sense RNA viruses, including the viral RdRp, remain at the forefront of drug-design strategies. Nucleoside analogs (NA) represent a promising class of RdRp inhibitors, and are currently used for the treatment of several other viral infections. NA prodrugs are metabolized in the host cell into active 5′-triphosphate forms that compete with natural nucleoside triphosphates (NTP) for incorporation into the viral RNA. This results in either chain-termination of viral RNA synthesis, or increases the viral mutation load to levels that can lethally alter the genetic make-up of the virus. Many details of NTP incorporation into viral RNA have been determined at the structural level following the pioneering work on the Reovirus RdRp^1^ (reviewed in ref. ^2^). However, CoVs stand out among RNA viruses for possessing an RNA-repair 3′-to-5′ exonuclease (ExoN, nsp14 stimulated by nsp10) able to excise mismatched bases as well as NAs incorporated into viral RNA, generally compromising the efficacy of these drugs^3–6^. Recently, the guanosine analog phosphoramidate prodrug AT-527 (Fig. 1a, left), was shown to act as a potent broad-spectrum anti-CoV inhibitor in a variety of cell lines^7^. It is now in phase III and II clinical trials for the treatment of COVID-19 and hepatitis C virus (HCV) infections^8^, respectively^9^ (www.clinicaltrials.gov/ct2/). AT-527 and its active 5′-triphosphate AT-9010 (Fig. 1a, right) carries a 2′-fluoro-2′-C-methyl modified ribose, identical to that of the clinically relevant anti-HCV uracil prodrug Sofosbuvir^10^.

The CoV genome contains ~30,000 nucleotides, about three times more than that of other significant human pathogenic +RNA viruses (see ref. ^11^ for review). Genome size reflects the presence of novel domains, many of which remain poorly characterized. Among these is the Nidovirus RdRp-Associated Nucleotidyltransferase (NiRAN) N-terminal domain of nsp12, just upstream of the RdRp^12^. The NiRAN is structurally related to the pseudo-kinase family of enzymes^13^, and has been shown to mediate the covalent transfer of nucleoside monophosphates (NMP) to viral cofactor proteins nsp7, nsp8 and nsp9, in a process known as NMPylation^14–16^. However, the role of this activity in the viral life-cycle remains unknown. The NiRAN has additionally been proposed to participate in the guanylyltransferase step of viral RNA capping^17^. Mutations in the NiRAN are lethal to the virus^12^, making it an attractive candidate for CoV-specific drug targeting.

Here, we show that AT-9010 inhibits two separate enzyme activities of the SARS-CoV-2 replicase/transcriptase complex. First, it acts as an immediate RNA chain terminator at the RdRp active site and provides partial resistance to excision by the nsp14/nsp10 ExoN. Second, it is a NMPylation inhibitor of nsp8 and nsp9 when bound into a deep pocket located at the NiRAN active site.

## Results

### *S*tructural studies of AT-9010 bound to the SARS-CoV-2 replicase:RNA complex

To investigate the AT-527/AT-9010 mechanism of action we performed cryo-EM studies with the SARS-CoV-2 minimal replication-transcription complex (RTC), comprised of nsp12 and essential cofactors nsp7 and nsp8^18,19^. The complex was formed in the presence of both AT-9010 and an annealed primer-template RNA. Image processing of single particles allowed the reconstruction of RTC:RNA:AT-9010 complexes at 2.98 Å resolution (Supplementary Table 1 and Supplementary Fig. 1).

The overall RTC conformation resembles previous structures, with one nsp12, one nsp7 and two nsp8 proteins (Fig. 1b)^20–22^. Both nsp12 (residues 4-929) and nsp7 (residues 2–73) are almost fully resolved, while the two nsp8s are mostly resolved (residues 38–191 and 43–192). As observed in ref. ^23^, the dsRNA stabilizes both nsp8 alpha-helical extensions in their N-terminus regions. The RdRp domain adopts a canonical right-hand fold, with fingers, palm and thumb subdomains (for review^2^). As for all viral RdRps, the active site is formed by five conserved motifs, A-E, located on the palm subdomain, with two additional motifs, F and G, located in the finger subdomains. The fully resolved N-terminus NiRAN is located alongside the palm domain of the RdRp^23,24^ (Fig. 1b).

### AT-9010 terminates the growing RNA chain

**Fig. 2:**
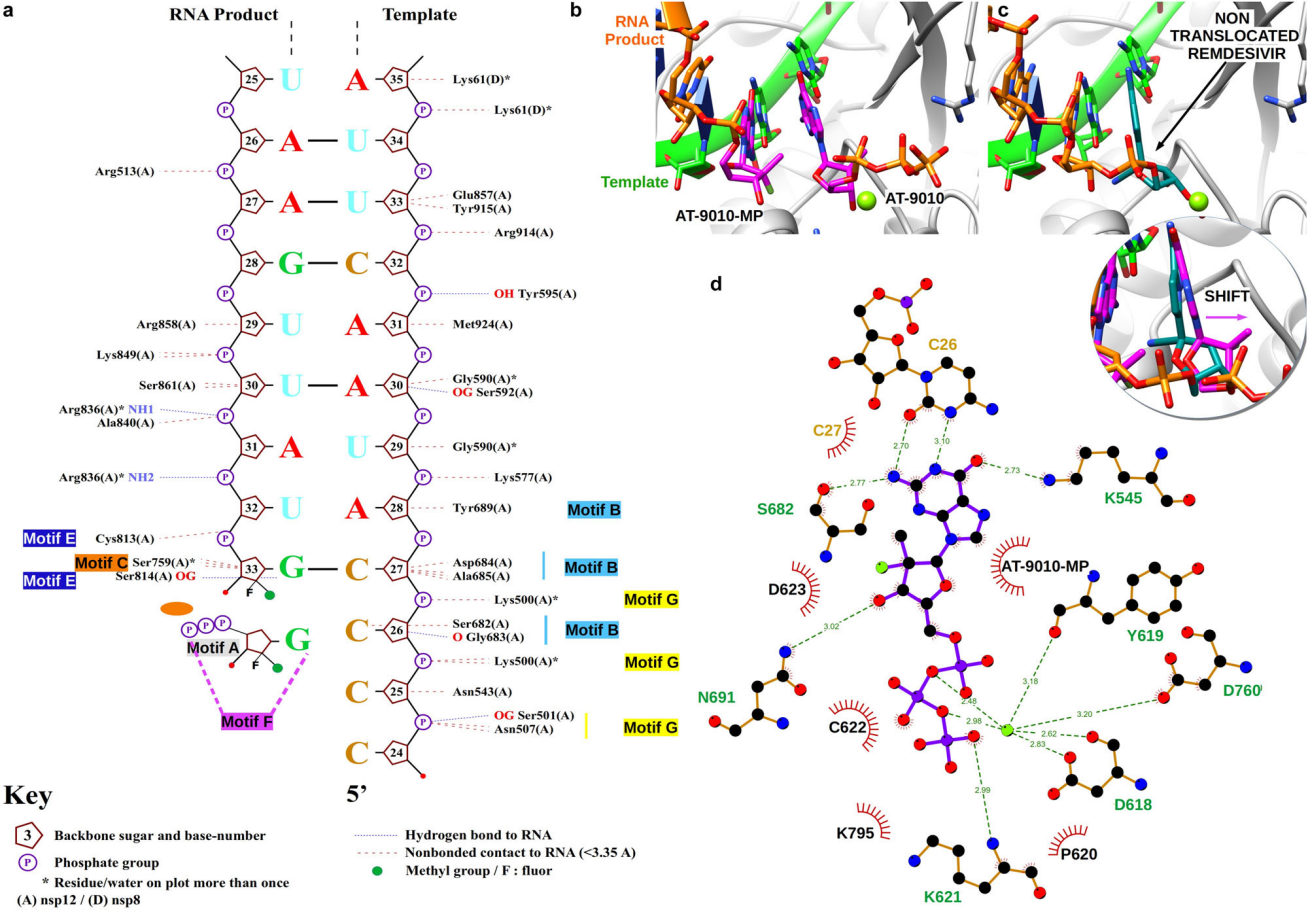
Structural basis for inhibition of the SARS-CoV-2 polymerase complex by AT-9010. **a** Nucplot molecular analysis with RNA, AT-9010 and nsp12. **b** Close up of RdRp catalytic site following AT-9010 incorporation. **c** Close up of RdRp catalytic site following remdesivir incorporation (PDB 7BV2). Both structures in panel **b** and **c** are in the same orientation, superimposed by least squares fit method (shown in circle). One AT-9010 5’-monophosphate (AT-9010-MP) is incorporated into the primer RNA strand, and terminates RNA elongation. The second AT-9010 molecule, coordinated by one ion, occupies the NTP-binding site in comparison, remdesivir is terminally incorporated, and untranslocated. Superimposition shows the incoming (−1) AT-9010 ribose group is shifted with the phosphates in a post-incorporation position. Ribbons are depicted as follows: nsp12, blue; nsp7, pink; nsp8_1_ and nsp8_2_, yellow and cyan, respectively; RNA, green sticks; AT-9010, magenta sticks. **d** Ligplot 2D analysis of the contacts of AT-9010 molecule with nsp12, and incorporated AT-9010-MP.

The 5′-end of the RNA template contains four consecutive cytidine bases (C24–C27), directing incorporation of AT-9010 into RNA (Figs. 1c, d and 2a). The 3′ end of the RNA primer, is stabilized by Ser814 bonding through a non-bridging oxygen of the last phosphodiester bond. AT-9010 is incorporated at the first position (+1), base pairing with C27 of the template strand (Fig. 1b, c and Supplementary Movie 1). A second, free AT-9010 occupies the NTP-binding site, (−1) position, but is not correctly poised for incorporation (Fig. 1c). Palm domain residues Asp618 and Asp760 in motifs A and C, respectively, coordinate a single magnesium ion, which interacts with oxygens of the α and β phosphates of the second AT-9010 (Fig. 2). Motif A and C acidic residues usually coordinate two metal ions^22^. The missing catalytic Mg^2+^ in the structure normally plays a critical role in the positioning of the 3′ end of the RNA primer and the incoming NTP.

### Chain-terminated RNA and next AT-9010 disrupts the catalytic site

The second, incoming (−1) AT-9010 guanine base is partially base-paired with cytosine (C26) of the template strand, and further stabilized by motif F Lys545 and motif B Ser682, two residues important during the fidelity check prior to incorporation (Figs. 1c and  2b–d)^25,26^. The α- and β- phosphates are coordinated by the single Mg^2+^, while the γ-phosphate is stabilized by motif A Lys621 and motif D Lys795 (Fig. 2d). The 3′-OH of the elongated primer is too far from the α-phosphate for incorporation (6.3 Å) and furthermore the metal is not correctly aligned for catalysis. Rather, we observe that AT-9010 has its α- and β-phosphates spatially overlapping the expected position of the leaving pyrophosphate (β- and γ-phosphates) (Fig. 2c), as judged by comparing with either superimposed enterovirus RdRp structures or incorporated Remdesivir by SARS-CoV-2 nsp12^22,27^.

The effect of the 2′-fluoro and 2′-C-methyl ribose modifications of the incorporated AT-9010 are as follows: The replacement of the 2′ hydroxyl by a fluoro group eliminates a stabilizing interaction with Ser759 of the SDD palm domain motif C. Comparison of the SARS-CoV-2 nsp12:RNA:Remdesivir and Favipiravir-ribose 5′-triphosphate cryo-EM structures with HCV NS5:RNA:Nucleotide ternary complexes^22,28,29^ shows a tight superimposition of catalytic residues, RNA backbone, and nucleotides/NAs except in the case of AT-9010 (Supplementary Fig. 2). The incoming AT-9010 ribose group is shifted in comparison to its expected position^22,28,30^ (Fig. 2c, d and Supplementary Fig. 2a, b, c). Its orientation is also different to that of pre-incorporated, (−1) translocated Sofosbuvir 5′-diphosphate and GS-9813 5′-diphosphate in HCV-RdRp structures^29^. As these two NAs carry the same 2′-fluoro-2′-C-methyl ribose as AT-9010, this demonstrates that this shift is neither dependent on the nature of the base nor on the ribose modification of the incoming NA 5′-triphophate (Supplementary Fig. 2). Rather, the hydrophobic 2′-methyl group of the incorporated AT-9010 ribose creates a repulsive hydrophobic-polar contact with the ribose ring oxygen of the incoming NA (or NTP) (Fig. 2b), causing chain-termination. Following 2′-fluoro-2′-C-methyl NA incorporation into RNA by any given viral RdRp, this ribose modification may well promote a universal mechanism of chain-termination.

### AT-9010 is a potent RNA chain terminator substrate

**Fig. 3:**
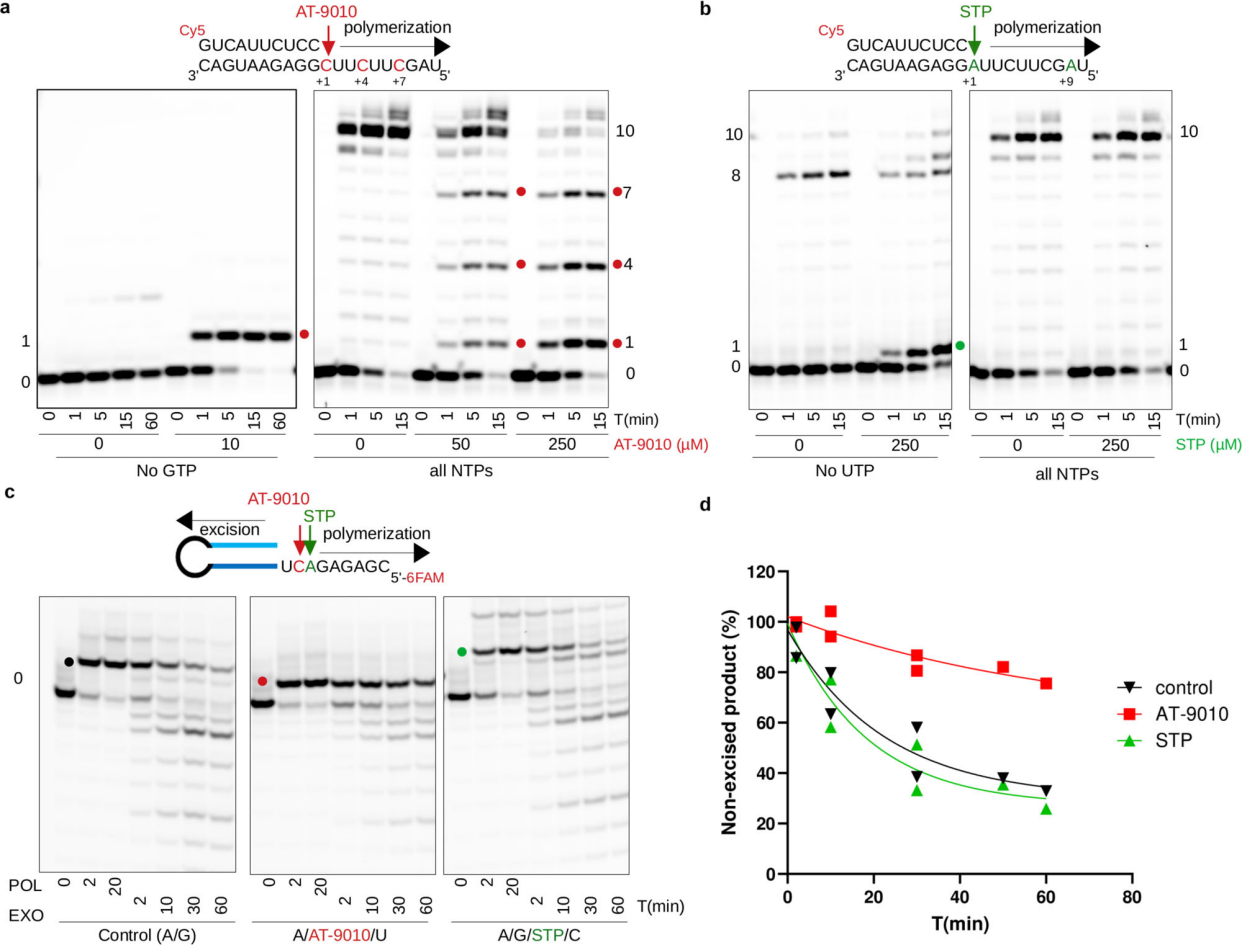
Incorporation of AT-9010 and Sofosbuvir triphosphate (STP) by the SARS-CoV-2 RTC, and excision by the SARS-CoV-2 nsp14/10 exonuclease complex (ExoN). **a** Timecourse of AT-9010 incorporation (red dots) as a substitute for GTP (left panel, 50 µM each of ATP, UTP, and CTP), or in competition with GTP (right panel, 50 μM each NTP) at indicated concentrations of AT-9010 (0–250 µM). Fold-preference for GTP over AT-9010 incorporation is calculated by comparing the amount of AT-9010 insertion (red dots) relative to full-length product at two concentrations and at three time-points. **b** Timecourse of Sofosbuvir triphosphate (STP) incorporation (green dots) as a substitute for UTP (left panel, 50 µM each of ATP, GTP and CTP), or in competition with UTP (50 μM each NTP) at indicated concentrations (0 or 250 µM). For both **a** and **b**, reactions were run at least in duplicate at multiple nucleotide/analog concentrations for two different RNA substrates, with consistent results. **c** Incorporation of ATP+GTP control (black, left panel), ATP + AT-9010 (red, middle panel) and ATP + GTP + STP (green, right panel) by the polymerase complex (POL), followed by excision time-course with the nsp14 ExoN (EXO). For both AT-9010 and STP experiments, the next templated nucleotide for incorporation was additionally added. **d** Quantitation of remaining product after ExoN excision shown in **c**, a representative gel of experiments done in duplicate for two separate RNAs. The RNA marked at position 0 on each gel corresponds to the size of the fluorescently labeled primer shown above each gel, prior to elongation. Source data are provided as a Source Data file.

We compared AT-9010 incorporation and elongation by the purified, recombinant SARS-CoV and SARS-CoV-2 RTCs using a heteropolymeric RNA primer:template pair, corresponding to the 3′ end of the SARS-CoV-2 genome^26^. In the absence of GTP, AT-9010 is rapidly incorporated into viral RNA causing immediate termination of RNA synthesis (Fig. 3a). Even at concentrations of 500 µM of ATP (the next templated nucleotide), elongation past the incorporated AT-9010 is not observed (Supplementary Fig. 3a). Consistent with structural analysis, this indicates that chain-termination is independent of the identity of the incoming NTP. Rather, the AT-9010 ribose modification causes a misalignment that prevents further elongation (Fig. 2). Importantly, in the presence of equimolar concentrations of GTP, AT-9010 acts as a competitive guanosine substrate, discriminated against only ~5-fold (Fig. 3b). We additionally compared AT-9010 incorporation with the structurally related, uracil analog Sofosbuvir triphosphate (STP). Although STP is able to be incorporated by nsp12 as a substitute for UTP, it is not competitive with UTP, even at 5-fold higher concentrations (Supplementary Fig. 3b).

In both human bronchial and nasal epithelial cells incubated with 10 µM of the AT-527 prodrug, intracellular concentrations of AT-9010 peak at ~700 µM and 240 µM, respectively^7^. Considering an average intracellular concentrations of GTP of ~500 µM^31^, it is expected that AT-9010 would be highly competitive for insertion into viral RNA, frequently terminating synthesis.

### RNA chain-terminated by AT-9010 shows resistance to ExoN removal

The stalling of the polymerase following insertion of a chain-terminating NA may allow excision by the proofreading 3′-to-5′ exonuclease nsp14/nsp10^6^ potentially dampening AT-9010 efficacy. Following incorporation into RNA, both AT-9010 and STP are excised by the SARS-CoV-2 ExoN (Fig. 3b, c). Interestingly, however, AT-9010 is ~4.3-fold more resistant to excision relative to an unmodified or STP-terminated RNA 3′-end (Fig. 3b, c). Given the highly competitive incorporation of AT-9010, this reduced ExoN-rate likely slows viral replication, even if complete excision may eventually be achieved.

### AT-9010 binds into the active site of the NiRAN domain

**Fig. 4:**
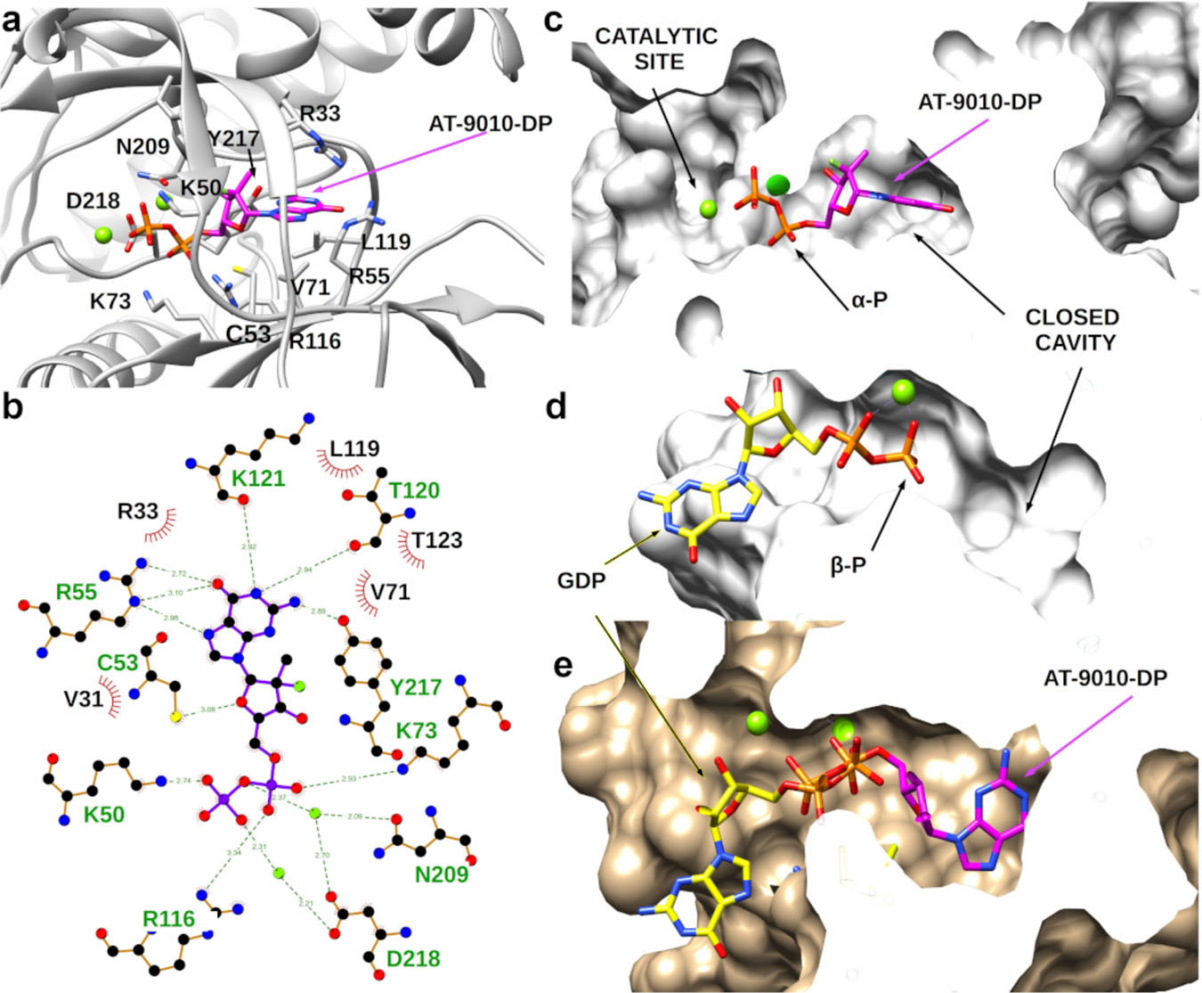
Structural basis for NiRAN inhibition of the SARS-CoV-2 by AT-9010. **a** Binding of AT-9010 5’-diphosphate (AT-9010-DP) in the NiRAN domain. Interacting residues are labeled and shown in stick representation. **b** Ligplot 2D representation of the detailed interactions of AT-9010-DP binding in the NiRAN cavity. **c** Surface representation of the NiRAN sliced orthogonally in Z showing AT-9010-DP engulfed in the cavity. **d** Surface representation of the NiRAN sliced orthogonally in Z showing GDP binding (PDB 7CYQ); Orientation is rotated by ~+90° compared to **c**. **e** Superimposition of GDP and AT-9010-DP showing the overlap of the α-phosphate of AT-9010 with the β-phosphate of GDP and Mg^2+^ in the two structures, same orientation as **d**. Color code is as follows: AT-9010, magenta sticks; GDP, yellow sticks; and Mg^2+^, light green.

A third AT-9010 is found in the N-terminal NiRAN domain of nsp12 (Figs. 1b and  4 and Supplementary Movie 2). Initial attempts to place the AT-9010 triphosphate group lead to a steric clash between a non-bridging oxygen of the γ-phosphate and the carboxyl group of Asp218. However, the density suggests the presence of an additional metal ion, as seen in the SelO pseudo-kinase structure^13^. Density and coordination distances between this ion, the AT-9010-β phosphate and the Asp218 carboxyl group are coherent, showing AT-9010 is in its diphosphate (DP) form—herein referred to as AT-9010-DP (Fig. 4b and Supplementary Fig. 4a). In support of the DP form, HPLC analysis of AT-9010 before and after incubation with either nsp12 wild-type (WT), or NiRAN mutant, or RdRp active-site mutant shows that the NiRAN domain mediates hydrolysis of the γ-phosphate. The presence of the DP form increases 2.3–2.8-fold with WT and RdRp active-site mutants, but not following incubation with the NiRAN active-site mutant (Supplementary Fig. 4b).

The NiRAN catalytic center contains a groove harboring two catalytic ions, coordinated by conserved residues Asn209 and Asp218 (Fig. 4c, e), leading to a narrow cavity. The α and β phosphates of AT-9010 are stabilized in the groove by the two ions and residues Lys50, Lys73 and Arg 116 (Fig. 4a). Intriguingly, the orientation of AT-9010-DP is strikingly different to that of ADP and GDP in existing NiRAN structures^17,32^, as well as other nucleotide-bound pseudo-kinase structures (Fig. S4a–c)^20,21^. Rather, the binding mode is reminiscent of the orientation of ATP bound to casein kinase^33^. The AT-9010-DP guanine base and modified ribose are accommodated in an inner narrow cavity (Fig. 4), which is unoccupied in existing NDP-NiRAN structures^17,32^. The guanine base is extensively stabilized by hydrophobic interactions and through hydrogen bonding with Arg55, Thr120 and Tyr217, residues which are conserved in CoV nsp12 sequences (Fig. 4a, b), but absent in other pseudo-kinases (Supplementary Fig. 5). The AT-9010-DP α- and β-phosphates are coordinated in the same groove as the phosphates of GDP and ADP in other NiRAN structures, but are in a flipped orientation, occupying the usual positions of the β- and α-phosphates, respectively^17,32^. AT-9010-DP has thus a unique binding mode, driven by both the hydrophobic nature of the cavity and modified ribose.

### AT-9010 inhibits nsp9 and nsp8 UMPylation

The NiRAN has previously been shown to mediate the covalent transfer of NMPs^13^ to various cofactor proteins^14–16^. In these studies, UTP was demonstrated to be the preferred substrate for cofactor labeling. However, our structural analysis shows that the AT-9010 guanosine base is well accommodated into the NiRAN. To determine whether AT-9010 is able to inhibit NiRAN-mediated transferase activity, we performed competition experiments measuring the efficiency of nsp9-UMPylation by both SARS-CoV and SARS-CoV-2 RTCs in the presence of increasing concentrations of AT-9010, or its uracil equivalent STP (Supplementary Fig. 6a). Both drugs inhibit nsp9 labeling at comparable levels for SARS-CoV and SARS-CoV-2 RTCs. When provided at equimolar concentrations to UTP, nsp9-UMPylation is inhibited ~85–90% by both STP and AT-9010 for the SARS-CoV-2 complex, showing both drugs outcompete UTP for NiRAN-binding. Nsp8 has additionally been shown to act as a target substrate for NiRAN-mediated UMPylation by the CoV RTC^14,16^. Interestingly, AT-9010 is ~4-5-fold more efficient at blocking nsp8 labeling than the uracil equivalent STP (Supplementary Fig. 6b). Furthermore, given the excess of nsp8 provided in the reaction, these inhibition data suggest that AT-9010 remains stably bound into the NiRAN active site, rather than being transferred to nsp8.

### AT-9010 is stably bound in the NiRAN active site

Thermal shift assays with nsp12 confirms that AT-9010 provides more thermodynamic stability than any other native nucleotide (Supplementary Fig. 6c). Comparison of NiRAN and RdRp active-site mutants (K73A and SAA, respectively) shows that this stability increase is provided by AT-9010 binding preferentially to the NiRAN, rather than the RdRp active site. Both GTP: and AT-9010:nsp12 complexes show an increased thermal stability compared with UTP or STP-bound complexes. Overall, and consistent with structural analysis, these results indicate that guanosine is a stable ligand of the NiRAN active site, with the 2′-fluoro-2′-C-methyl ribose modification of AT-9010 likely allowing insertion into the pocket and/or providing additional stability. In contrast, the binding of UTP is weak and putatively transient, likely to facilitate transfer to CoV nsps and possibly other targets.

## Discussion

AT-9010 is efficiently incorporated into viral RNA, causes immediate chain-termination, and shows resistance to excision. Additionally, the superior affinity of guanosine analogs for the NiRAN as well as its deep AT-9010-binding pocket uncovers a specific druggable site of remarkable interest for anti-coronavirus research. The respective inhibition impact of AT-9010 on each of these activities is unknown. We note that the precise role of the NiRAN in the viral life-cycle is still somewhat speculative, but mutagenesis has shown that its NMPylation activity is essential^12^. Nucleotide transferase enzymes are required for a range of reactions including, e.g., RNA capping, DNA/RNA ligation, and priming of RNA synthesis, making it possible that the NiRAN is involved in various viral processes. Currently, there is some evidence for both guanylyltransferase and RNA-priming activity for the NiRAN domain of SARS-CoV-2^16,17^. Here, we show through both structural and biochemical analysis that AT-9010 binds preferentially, and stably in the NiRAN active-site pocket. Both of the reported NMPylation events (nsp8 and nsp9) are blocked efficiently by AT-9010. Therefore, any downstream activities dependent on this nucleotidylation event, or dependent on NiRAN-NTP binding in general (including capping, DNA/RNA ligation, or priming of RNA synthesis) would be blocked.

We note that the serendipitous simultaneous targeting of RdRp and NiRAN essential activities by the same drug adds momentum to the use of inhibitors against COVID-19 and the SARS-CoV-2 RTC, which remarkably has remained genetically stable since the beginning of the pandemic. Such AT-9010 pleiotropic action should attenuate the chance of simultaneous resistance mutations, and be an important asset in the control of the expanding genetic pool of SARS-CoV-2 variants observed in the current pandemic.

## Methods

### 5′-triphosphate nucleosides

AT-9010, and 5′-triphosphate 2′-fluoro-2′-C-methyl uridine (Sofosbuvir 5′-TP, STP) were provided by NuBlocks LLC, Oceanside, CA, USA. Other NTPs were HPLC grade, purchased from GE Healthcare.

### Expression and purification of SARS-CoV proteins

The SARS-CoV and SARS-CoV-2 sequences used in this study are from the Tor2 (NCBI ref. seq.: NC_004718.3) and Wuhan-Hu-1 (NCBI ref. seq.: YP_009724389.1) isolates, respectively. Unless specified ortherwise, a N-terminus 6His tag was fused to a TEV cleavage site ENLYFQG on which the last glycine was replaced by the first amino acid of the coding sequence of the gene of interest.

SARS-CoV proteins 6His(TEV)nsp7 and 6His(TEV)nsp8 were expressed under the control of a T5-promoter in pQE30 vectors in Escherichia coli (*E. coli*) NEB Express C2523 cells (New England Biolabs) carrying the pRare2LacI (Novagen) plasmid. Protein was expressed overnight at 17 °C (with 100 µg/mL ampicillin and 17 µg/mL Chloramphenicol), following induction with 100 µM IPTG at an OD_600_ = 0.5–0.6. Cells were lysed by sonication in lysis buffer (50 mM Tris-HCl pH 8, 300 mM NaCl, 10 mM imidazole, supplemented with 20 mM MgSO_4_, 0.25 mg/mL Lysozyme, 10 μg/mL DNase and 1 mM PMSF) and protein was purified through affinity chromatography with TALON® Superflow™ cobalt-based IMAC resin (Cytiva). A wash step with buffer supplemented with 500 mM NaCl was performed prior to elution with 200 mM imidazole. The affinity tag was removed via overnight cleavage with TEV protease (1:10 w/w ratio to TEV:protein) in a dialysis buffer containing no imidazole and supplemented with 1 mM DTT. Cleaved protein was re-purified through a second cobalt column to remove the histidine-labeled TEV protease, and further purified with size exclusion chromatography (Cytiva Superdex S200) in a final buffer of 25 mM HEPES pH 8, 150 mM NaCl, 5 mM MgCl_2_ and 5 mM TCEP.

SARS-CoV nsp12-8His was expressed from a pJ404 vector in *E. coli* strain BL21/pG-Tf2 (Takara 9124), in the presence of ampicillin (100 µM/mL) and Chloramphenicol (17 µg/mL). Expression was induced at OD_600_ = 0.5–0.6 with 250 µM IPTG and 5 ng/mL of tetracycline for induction of chaperones proteins (groES-groEL-tig), and left overnight at 23 °C at 220 rpm. Cells were lysed over 45–60 mins at 4 °C, in a buffer containing 50 mM Tris pH 8, 300 mM NaCl, 5 mM MgSO_4_, 10% glycerol, 1% CHAPS, supplemented with 5 mM 2-mercaptoethanol, 0.5 mg/mL Lysozyme, 10 μg/mL DNase, 1 mM PMSF, 0.2 mM benzamidine. NaCl was gradually added to a final concentration of 1 M, to aid in the removal of contaminating nucleic acids. Following centrifugation (30,000 × *g* for 30 min), the supernatant was diluted to reduce NaCl concentration to 300 mM. Protein was purified using cobalt-based IMAC resin TALON® Superflow™ (Cytiva), washing three times with wash buffer (50 mM Tris pH 8, 10% glycerol) with alternating NaCl concentration (300 mM, 1 M, 300 mM) before elution with 200 mM imidazole. Protein was further purified through size exclusion chromatography (Cytiva Superdex S200) in the same final buffer as nsp7 and nsp8 proteins, supplemented with 10% glycerol. Concentrated aliquots of nsp12, 7, and 8 were flash-frozen in liquid nitrogen and stored at –80 °C.

SARS-CoV nsp9 was expressed with self-cleavable ubiquitin fused at its N-terminus and 6His at its C-terminus under the control of a Tet-promoter in a pASK vector in *E. coli* NEB Express C2523 cells carrying the pCG1 plasmid (New England Biolabs). Protein was expressed overnight at 20 °C (with 50 µM/mL kanamycin, 17 µg/mL chloramphenicol), following induction with 200 µg/L anhydrotetracycline at an OD_600_ = 0.6–0.7. Cells were incubated in lysis buffer (50 mM HEPES pH 7.5, 300 mM NaCl, 10 mM midazole, 5 mM MgSO_4_, 1 mM of β-mercaptoethanol, 0.25 mg/mL Lysozyme, 10 μg/mL DNase, 0.1% triton and 1 mM PMSF) and lysed by sonication. Protein was purified first through affinity chromatography with HisPur Cobalt resin (Thermo Scientific), eluted in 100 mM imidazole, then through size exclusion chomatography (GE Superdex S200) in a final buffer of 50 mM HEPES pH 7.5, 300 mM NaCl, 5 mM MgCl_2_ and 1 mM of β-mercaptoethanol.

SARS-CoV protein nsp10 carrying a non-cleavable 6His tag in N-terminus was expressed under the control of a Tet-promoter in a pASK vector in *E. coli* NEB Express C2523 cells (New England Biolabs) carrying the pRare2LacI (Novagen) plasmid. Protein was expressed overnight at 17 °C (with 50 µM/mL kanamycin, 17 µg/mL chloramphenicol), following induction with 200 µg/L tetracycline at an OD600 = 0.6–0.7. Cells were incubated in lysis buffer (50 mM HEPES pH 7.5, 300 mM NaCl, 10 mM imidazole, 5 mM MgSO_4_, 1 mM of β-mercaptoethanol, 0.25 mg/mL Lysozyme, 10 μg/mL DNase, 0.1% Triton and 1 mM PMSF) and lysed by sonication. Protein was purified first through affinity chromatography with HisPur Cobalt resin (Thermo Scientific), eluted in 100 mM imidazole, then through size exclusion chomatography (GE Superdex S200) in a final buffer of 50 mM HEPES pH 7.5, 300 mM NaCl, 5 mM MgCl_2_ and 1 mM of β-mercaptoethanol.

SARS-CoV nsp14 carrying a non-cleavable 6His tag in N-terminus was expressed from a pDEST14 vector in *E. coli* strain NEB Express C2566 cells (New England Biolabs) carrying the pRare2 plasmid, in the presence of ampicillin (100 µM/mL) and chloramphenicol (17 µg/mL). Protein expression was induced at an OD_600_ = 0.8 with 2 µM IPTG, and left overnight at 17 °C with shaking. Cells were lysed by sonication in a buffer containing 50 mM HEPES pH 7.5, 500 mM NaCl, 20 mM imidazole, supplemented with 0.25 mg/mL Lysozyme, 10 μg/mL DNase and 1 mM PMSF. The protein was purified through affinity chromatography with HisPur Cobalt resin (Thermo Scientific), washing with an increased concentration of salt (1 M NaCl), prior to elution in buffer supplemented with 250 mM imidazole. The protein was further purified by a size exclusion chomatography (GE Superdex S200) in a final buffer of 10 mM HEPES pH 7.5, 150 mM NaCl.

SARS-CoV-2 proteins were either purchased from Biortus (en.wuxibiortus.com) or purified using the following protocols.

For Cryo-EM, the gene of the full-length SARS-CoV-2 nsp12 (residues 1-932) was synthesized with codon optimization (General Biosystems) and cloned into pFastBac1 baculovirus expression vector. An additional peptide (MHHHHHHHHWSHPQFEKENLYFQG) was added to the N-Terminus of nsp12. *Spodoptera frugiperda* (*Sf21*) cells expressing the target protein were collected 48 h after infection at 27 °C and were centrifuged at 4300 × *g* for 10 min. Pellets were resuspended in lysis buffer (50 mM Tris-HCl, pH 8.0, 500 mM NaCl, 5% glycerol, 2 mM MgCl_2_, Complete Protease Inhibitor Tablet) and homogenized with High-Pressure Homogenizer at 4 °C. Cell lysate was centrifuged at 27,000 × *g* for 60 min at 4 °C. The fusion protein was first purified by Strep-Tactin (Strep-actin®XT) affinity chromatography and the tag was removed by incubation of TEV protease overnight at 4 °C after elution. The protein was reloaded onto a Heparin HP column after buffer exchanged by ultrafiltration tubes to buffer A (50 mM Tris-HCl, pH 8.0, 150 mM NaCl, 5% glycerol, 2 mM MgCl_2_). Flow through was collected and loaded on to a HiLoad 16/600 Superdex 200 pg column (GE healthcare) equilibrated in 10 mM Tris-HCl, pH 8.0, 500 mM NaCl, 2 mM MgCl_2_. Purified nsp12 was concentrated to 6.86 mg/mL with ultrafiltration tubes and stored at –80 °C.

The gene of SARS-CoV-2 nsp7 (residues 1-83) possessing a C-terminal Avi-6His tag (GLNDIFEAQKIEWHEHHHHHH) was cloned into a modified pET-32a vector. BL21 (*E. coli*, T7 Express) containing the plasmid were grown to an OD600 of 0.6 at 37 °C, and protein was expressed at 15 °C for 16 h after the addition of isopropyl β-D-1-thiogalactopyranoside (IPTG) to a final concentration of 0.5 mM. The cells were harvested then resuspended in buffer B (50 mM Tris-HCl, pH 8.0, 500 mM NaCl, 5% glycerol, 10 mM imidazole). Cells were disrupted by a High-Pressure Homogenizer at 4 °C. The insoluble material was removed by centrifugation at 27,000 × *g*, 60 min at 4 °C. The fusion protein was purified by Ni-NTA (Novagen, USA) affinity chromatography followed by a Superdex HiLoad 16/600 Superdex 75 pg column (GE Healthcare, USA) in buffer C (10 mM Tris-HCl, pH 8.0, 150 mM NaCl). Purified nsp7 was concentrated to 7.27 mg/mL with ultrafiltration tubes and stored at –80 °C.

The gene of SARS-CoV-2 nsp8 (residues 1–198) was cloned into a modified pET-28a vector containing an N-terminal His6-flag-tag with a TEV cleavage site (MHHHHHHDYK DDDDKENLYFQG) for expression in *E. coli*. Nsp8 was expressed in the same way as that for nsp7. Cells were harvested then resuspended in buffer D (50 mM Tris-HCl, pH 8.0, 500 mM NaCl, 5% glycerol). Cells were lysed using a High-Pressure Homogenizer at 4 °C. Cell lysate was clarified by centrifugation at 27,000 × *g*, 60 min at 4 °C. Supernatant was applied onto a Talon affinity chromatography column and the tag was removed by on-column cleavage overnight using TEV protease. The mixture was buffer exchanged to buffer D with ultrafiltration tubes and reloaded onto a His-chelating NTA column again to remove the His tag and TEV protease. Target protein was further purified by passage through a HiLoad 16/600 Superdex 75 pg (GE Healthcare, USA) in buffer E (20 mM Tris-HCl, pH 8.0, 200 mM NaCl, 5% glycerol). The fractions near the maximum height of the peak were combined and further purified by a Mono Q 10/100 GL column (GE Healthcare, USA). Purified nsp8 was buffer exchanged to buffer E with ultrafiltration tubes, then concentrated to 11.63 mg/mL and stored at –80 °C.

For enzyme studies, SARS-CoV-2 proteins 6His(TEV)nsp7, 6His(TEV)nsp8, and nsp9-6His were obtained from *E.coli* expression clones. The SARS-CoV-2 sequences replaced those of SARS-CoV in cognate vectors. For SARS-CoV-2 nsp10 and nsp14, genes were cloned into the pET28a(+) vector with a 6His tag fused to the TEV cleavage sequence as described above; The proteins were purified following the same protocol as SARS-CoV 6His(TEV)nsp7 and 6His(TEV)nsp8, omitting hloramphenicol in the culture medium. Unlike nsp7 and nsp8, which always had their tags removed, nsp12 was used with or without 6His tag without noticeable different properties. Likewise, no noticeable differences were noted between SARS-CoV and SARS-CoV2 for their RTC or ExoN activities.

### Assembly of the extended nsp12-nsp7-nsp8-RNA complex for Cryo-EM

To assemble the Extended RdRp complex, nsp12 was incubated with nsp7 and nsp8 at 4 °C for 3 h with a molar ratio of 1:3:6 in a buffer containing 25 mM Tris-HCl, pH 8.0, 50 mM NaCl, 5 mM MgCl_2_, 4 mM DTT. Then the mixture was purified by Mono Q 5/50 GL ion-exchange chromatography (GE Healthcare, USA), resulting in a stable nsp7-nsp8-nsp12 complex. The protein complex was desalted to buffer F (25 mM Tris-HCl, pH 8.0, 100 mM NaCl, 5 mM MgCl_2_, 4 mM DTT). Purified RdRp complex was buffer exchanged to 25 mM Tris-HCl, pH 8.0, 100 mM NaCl, 5 mM MgCl_2_, 4 mM DTT and concentrated to 10 mg/mL for Cryo-EM experiments. A 30-mer oligoribonucleotide template (5′-CCCCCCCCCCAUAACUUAAUCUCACAUAGC-3′) and a 20-mer oligoribonucleotide primer (5′-GCUAUGUGAGAUUAAGUUAU-3′) were chemically synthesized by GenScript. They were annealed by heating an equimolar solution in RdRp buffer to 95 °C and gradually cooling to 4 °C. The annealed RNA scaffold was incubated with the nsp12-nsp7-nsp8 complex for 30 min at 4 °C with a molar ratio of 2:1 to form the nsp12-nsp7-nsp8-RNA complex. AT-9010 with a final concentration of 1 mM was added subsequently for compound incorporation.

### Cryo-EM sample preparation and data collection

In total, 3 µL of protein solution at 5 mg/mL (with 0.025% DDM) was applied onto a glow-discharged holey carbon grid (Quantifoil, 300 mesh, R1.2/1.3). Excess samples were blotted for 5.0 s with a blotting force of 3, then the remaining solution was vitrified by plunging into liquid ethane using a Vitrobot Mark IV (Thermo Fischer Scientific) at 4 °C and 100% humidity. Cryo-EM data were collected with a 300 keV Titan Krios electron microscope (Thermo Fisher Scientific, USA) equipped with a K3 direct electron detector (Gatan, USA) operating in a super-resolution counting mode. All movies were automatically recorded using SerialEM^34^ at a magnification of 105 K, with a physical pixel size of 0.83 Å. A total dose of 80.5 e-/Å^2^ was fractionated into 50 frames. 7,459 movie micrographs were collected with a defocus range from –1.5 to –2.5 µm, and the slit width of Gatan Quantum GIF energy filter (Gatan, USA) was set to be 20 eV. Statistics for data collection and refinement are shown in Supplementary Table 1.

### Cryo-EM image processing

All dose-fractioned movies were motion-corrected with Relion′s own implementation. CTF estimation, 2D classification, 3D classification and refinements were all performed in cryoSPARC. A total of 2,410,466 particles were auto-picked using blob picker and extracted with a box size of 320 pixels. 248,401 particles were selected after three rounds of 2D classification based on the complex integrity. This particle set was used for Ab-Initio reconstruction with three classes, which were then used as 3D volume templates for heterogeneous refinement. The 3D volume corresponding to the intact nsp12-nsp7-nsp8-RNA complex was used for creating 100 2D projections, which were then used as templates for template-based particle picking. Approximately 3,640,595 particles were picked from a set of 3622 micrographs filtered based on fitted resolution better than 5 Å as estimated by CTFFIND4, using template picker. Particles were extracted with a box size of 360 pixels. A total of 234,421 particles were selected after four rounds of 2D classification based on the complex integrity. This particle set was used for Ab-Initio reconstruction with three classes, followed by heterogeneous refinement. A subset of 181,669 particles from the class with good features was subjected to Homogeneous Refinement, Local Refinement and Non-uniform Refinement, resulting in a 2.98 Å map.

### Model building and refinement

To build the model of nsp12-nsp7-nsp8-RNA complex, the structure of SARS-CoV-2 nsp12-nsp7-nsp8-RNA complex (from PDB 7CYQ), with one nsp9 and nsp13 removed) was placed and rigid-body fitted into the Cryo-EM map using UCSF Chimera. The model was manually built in Coot^35^ with the guidance of the Cryo-EM map, and in combination with real space refinement using Phenix^36^. The model validation statistics are shown in Supplementary Table 1.

### HPLC analysis of nsp12-AT-9010 products

Wild-type nsp12 of SARS-CoV and SARS-CoV-2, as well as SARS-CoV NiRAN (K73A) and RdRp active-site mutants (SDD → SAA) were incubated with equimolar concentrations of AT-9010 triphosphate for one hour at 37 °C in a reaction buffer containing 50 mM HEPES, pH 7.5, 1 mM DTT and 1 mM MnCl. Reactions were heat inactivated at 70 °C for 10 min to promote dissociation of bound AT-9010 and products were purified through micocon spin columns (molecular weight cutoff 10,000). In parallel, a mock reaction without enzyme was performed under analogous conditions. Products were separated on HPLC C18 columns under a water: acetonitrile gradient to separate tri-, di- and mono-phosphorylated versions of AT-9010, UV detected and quantitated by determining peak surface areas.

### Primer-dependent polymerization and excision assays

Primer (5′cy5-GUCAUUCUCC-3′) and template (5′-UAGCUUCUU(A/C)GGAGAAUGAC-3′) RNA pairs, corresponding to the 3′ end of the SARS-CoV genome, were annealed at a molar ratio of 1:1.5 in 110 mM KCl at 70 °C for 10 min, then cooled slowly to room temperature over several hours. Hairpin RNAs were synthesized by Integrated DNA Technologies (Coralville, IA). The active RTC was formed by first incubating nsp7 and 8 together at equimolar concentrations (100 µM) for 30 min at room temperature. Nsp12, extra nsp8 and protein gel filtration buffer were added to form a final complex consisting of nsp12:7:8 at a 1:3:6 ratio, with 10 µM nsp12. The complex was further incubated for 10 mins at room temperature, then preincubated with RNA in a pre-mix containing 20 mM HEPES pH 7.5, 50 mM NaCl, 5 mM MgCl_2_. For single nucleotide incorporation assays, reactions were initiated with 50 µM (final concentration) of all AT-9010 or STP, with or without the following nucleotide (ATP). Final reaction concentrations were 0.5 µM nsp12, 0.4 µM RNA. Reactions were quenched after indicated time-points with 5X volume of FBD stop solution (formamide, 10 mM EDTA). For AT-9010–GTP competition experiments, protein-RNA complexes were preincubated as described above, and initiated with either all four NTPs, or with only CTP, UTP and ATP (50 µM each NTP), supplemented with various concentrations of AT-9010 (10–250 µM). To calculate the discrimination between AT-9010 and GTP, the AT-9010 product band (from 50 or 250 µM concentrations) was compared with the sum of fractions of product bands derived from GTP incorporation at three time-points. Discrimination was corrected to account for concentration difference between AT-9010 and GTP. For analog excision, polymerization reactions were performed on hairpin RNAs (5′-6-FAM-GCUUACGAGAAUGACAAAAGUCAUUCU-3′ and 5′-6FAM-GAAAGGUCUCGCGCCCGGGUAAUCGGGCGCGAGA-3′) in the same conditions as described above (labeled RTC, 2′ and 20′), then stopped by heating at 70 °C for 10 min. The hairpin was re-annealed at 30° for >30 min, and 50 nM nsp14/nsp10 (1:5) were added for time-course reactions (labeled Exo, 2′, 10′, and 60‘). Reaction products were separated using denaturing polyacrylamide gel electrophoresis (20% acrylamide, 7 M urea), visualized using a Typhoon FluorImager, and analyzed with ImageQuant software. Percent excision was calculated by dividing the intensity/volume of the band of interest (unexcised product) by the sum of all bands in the lane, to account for loading error. Excision percentage was plotted over time using GraphPad Prism, and fitted using a one-phase decay to obtain the reaction rate. The reaction was run in triplicate and in duplicate using two different hairpin RNAs.

### Nucleotidyl transferase inhibition reactions

Standard nucleotide-transfer reactions were performed at 37 °C in a buffer containing 20 mM HEPES pH 7.5, 1 mM DTT, 2 mM MnCl2, 1 µCi α^32^P-UTP, 30 mM NaCl and a 5 µM constant concentration of cold UTP, in competition with increasing concentrations of AT-9010 and STP (0.5–300 µM). Final protein concentrations of 1 µM nsp12 with 2 µM nsp9 or 5 µM nsp8 were used. Reactions were stopped after 15 min incubation, and analyzed on 15% sodium dodecyl sulphate–polyacrylamide gel electrophoresis gels, stained with InstantBlue for total protein and exposed for 2 h—overnight to reveal radiolabelled proteins. Nsp9 and Nsp8-UMPylation was calculated as a percentage relative to the no-inhibitor controls. Reactions were performed in triplicate with 6–7 concentrations of each NA.

### Thermal-shift assays

The influence of compound binding on protein stability was measured by Themofluor assay, using a CFX Connect BioRad real-time PCR machine. Reactions were run in thin-walled, 96-well plates, in a buffer composed of 10 mM HEPES pH 7.4, 150 mM NaCl, 5 mM MgCl_2_ and 0.5 mM MnCl_2_, in a final reaction volume of 20 µL. Compounds and NTP concentrations were fixed at 100 µM, with a final protein concentration of 2 µM (nsp12 WT and mutants) SYPRO Orange dye was used at a five-fold final concentration. Melting-temperature (*T*_m_) values given are the average and standard deviation of three independent experiments.

### Reporting summary

Further information on research design is available in the Nature Research Reporting Summary linked to this article.

## Supplementary information

Supplementary Information

Peer Review File

Reporting Summary

Supplementary Table 1

Supplementary Movie 1

Supplementary Movie 2

## Data Availability

The coordinates and structure factors for SARS-CoV-2 nsp7:nsp8:nsp12:RNA:AT-9010 quaternary structure have been deposited in the PDB with the following accession codes: 7ED5 and the EMDB: EMD-31061, respectively. Source data are provided with this paper.

## Source data

Source Data
