# Supplementary material for: A dual mechanism of action of AT-527 against SARS-CoV-2 polymerase
Source: Nat Commun. 2022 Feb 2;13:621. doi: 10.1038/s41467-022-28113-1 (PMC8810794; doi:10.1038/s41467-022-28113-1)
Supplement: Supplementary file 1 — Supplementary Information [file 41467_2022_28113_MOESM1_ESM.pdf]

Supplementary Information

Supplementary Table 1. Cryo-EM data collection, refinement, and validation statistics

Nsp12-nsp7-nsp8 complex	RNA and AT-9010 bound PDB 7ED5; EMDB: EM-31061
Data collection and Processing (for each dataset):	
Microscope/ Voltage (keV)	Titan Krios/300
Camera/ Magnification	Gatan K3 Summit/105,000
Pixel size at detector (Å/pixel)	0.83
Total electron exposure (e^-/Å^2)	80.5
Number of frames collected during exposure	50
Defocus range (μm)	-1.5 ~ -2.5
Phase plate (if used)	N/A
- phase shift range (in degrees)	N/A
- number of images per phase plate position	N/A
Automation software	SerialEM
Tilt angle	0
Energy filter slit width (eV)	20
Micrographs collected (no.)	7,459
Micrographs used (no.)	5,609
Total extracted particles (no.)	3,640,595
For each reconstruction:	
Refined particles (no.)/ Final particles (no.)	181,669/181,669
Point-group or helical symmetry parameters	C1
Resolution (FSC 0.143, Å)	2.98
Resolution range (local, Å)	2.7-3.3
Map sharpening B factor (Å^2)	82.7
Map sharpening methods	cryoSPARC v2.15.0
Model composition:	
Protein/ Ligands/ RNA	1302/8/44
Model Refinement:	
Refinement package	PHENIX-1.19_4085
- real or reciprocal space	real space
Model-Map CC	0.84
Model resolution (Å)	3.14
FSC threshold	0.5
B factors (Å^2)	
Protein residues	69.09
Ligands	67.51
RNA	134.15
R.m.s. deviations from ideal values	
Bond lengths (Å)	0.002
Bond angles (°)	0.538
Validation:	
MolProbity score	1.9
CaBLAM outliers	5.13
Clashscore	7.89
Poor rotamers (%)	0.09
C-beta deviations	0.00
EMRinger score (if better than 4 Å resolution)	3.32
Ramachandran plot	
Favored (%)	92.5
Outliers (%)	0.31

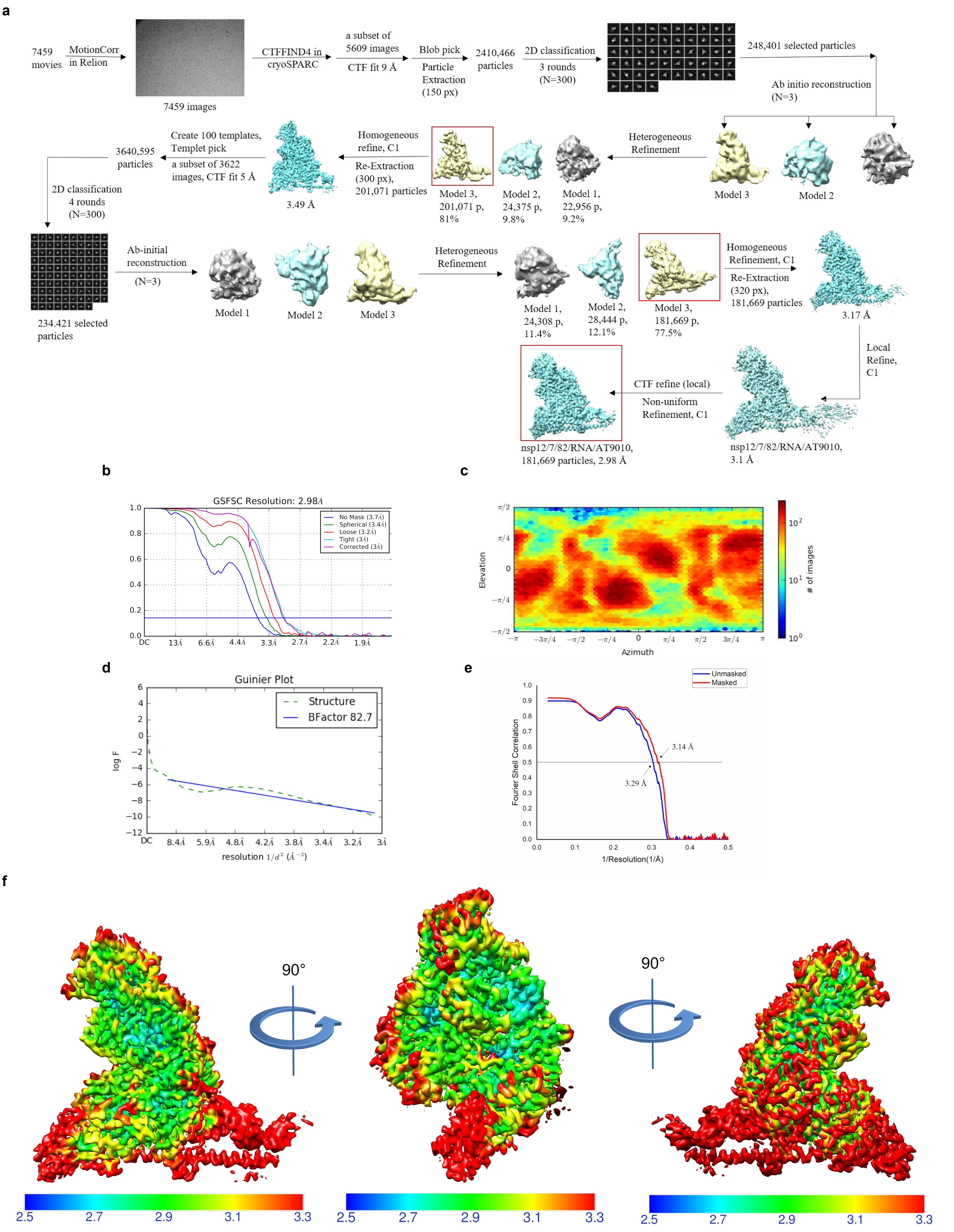

Supplementary Fig. 1. Cryo-EM data processing summary. (a) The flowchart shows the main steps in data processing, from particle picking, through classification, to final maps. A selected subset of the initial reference-free 2D class averages and all the intermediate 3D class averages computed during the processing of this dataset are shown. All 3D class averages, selected for subsequent rounds of processing, are boxed in red, and the percentage of number of particles in each of these is shown. Further attempts to process the discarded classes are omitted from this chart for clarity, as these data did not contribute to the final particle set.

(b-e) Map and model quality of the nsp7:$(nsp8)_2$:nsp12:RNA:AT-9010 complex. (b) FSC curves for the final reconstruction. The resolution of the map is 2.98 Å as calculated by the gold-standard FSC 0.143 criterion. (c) Viewing direction distribution of the final reconstruction. (d) The B-factor used for map sharpening is -82.7 Å2 as calculated by the Guinier Plot. (e) Model vs. map FSC curves. The resolution of the model is calculated to be 3.14 Å curves. The resolution of the model is calculated to be 3.14 Å based on the masked map. The local resolution for the RdRp active site and NiRAN domain active site is around 2.7-3.1 Å. (f) Local resolution distribution of the nsp7:nsp8:nsp12:RNA:AT-9010 complex calculated in cryoSPARC. Three views of the maps are shown rotated by 90° increments.

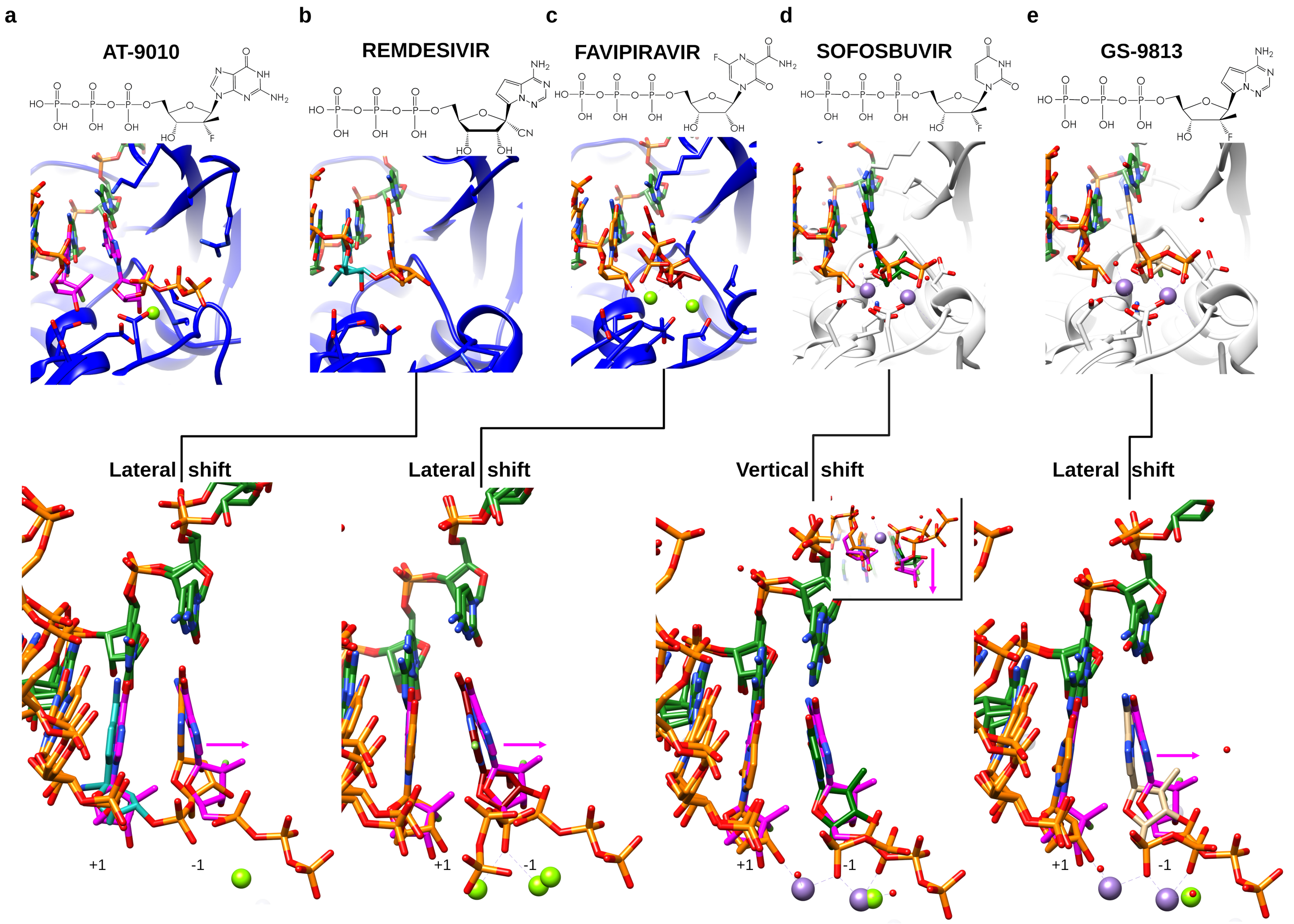

Supplementary Fig. 2. The effect of AT-9010 incorporation on the position of incoming NTP/NAs. From (a) to (e) all structures are in same orientation and superimposed by least squares fit method. (a) Zoom on RdRp catalytic site : One AT-9010 5'-monophosphate (AT-9010-MP) is incorporated into the primer RNA strand, and terminates RNA elongation. The second AT-9010 (-1), coordinated by one ion, occupies the NTP binding site. The position of the incoming (-1) AT-9010 is compared in panels (b) to (e), with superimposition shown below for each structure (AT-9010 in magenta). (b) GMP (orange) incorporated at the 3' end of the product RNA, following Remdesivir (cyan) incorporation in SARS-CoV-2 nsp12 (PDB: 7C2K, doi:10.2210/pdb7C2K/pdb). The GMP is not translocated following incorporation, and thus occupies the usual NTP binding site (-1) position. Superimposition shows the ribose of the (-1) AT-9010 is twisted in comparison to GMP, with its phosphates in a post-incorporation position normally occupied by PPi. (c) Favipiravir (dark red) occupies the NTP binding site in pre-incorporation (-1) position, coordinated by 2 Mg2+ ions, in SARS-CoV-2 nsp12 (PDB: 7AAP, doi:10.2210/pdb7AAP/pdb). Superimposition of the AT-9010 (-1) with Favipiravir shows ribose is shifted laterally, and phosphates are not in position for incorporation. (d) Sofosbuvir 5'-diphosphate (dark green) in the HCV RdRp occupies the (-1) NTP binding position, coordinated by 2 ions (PDB: 4WTG, doi:10.2210/pdb4WTG/pdb). The ribose of AT-9010 (-1) is shifted down (vertically) due to the methyl group of the incorporated AT-9010-MP, while the two bases superimpose well. (e) GS-9813 5'-diphosphate (beige) in the HCV RdRp occupies the (-1) NTP binding position, coordinated by 2 ions (PDB: 5UJ2, doi:10.2210/pdb5UJ2/pdb). The AT-9010 and GS-9813 bases do not superimpose, and the ribose of AT-9010 is shifted laterally due to the methyl group of the already incorporated AT-9010. For (d) and (e), the phosphates of the NAs are extensively coordinated by the metallic ions, and face towards the RNA product ready for incorporation. In contrast, the phosphates of AT-9010 occupy the site of the PPi product, and therefore cannot be incorporated. Color code is as follow: template RNA, green; RNA product, orange; $Mg^{2+,}$ light green; $Mn^{2+,}$ grey. RdRp are shown in ribbons/sticks: SARS-CoV-2 nsp12, blue; NS5, HCV white.

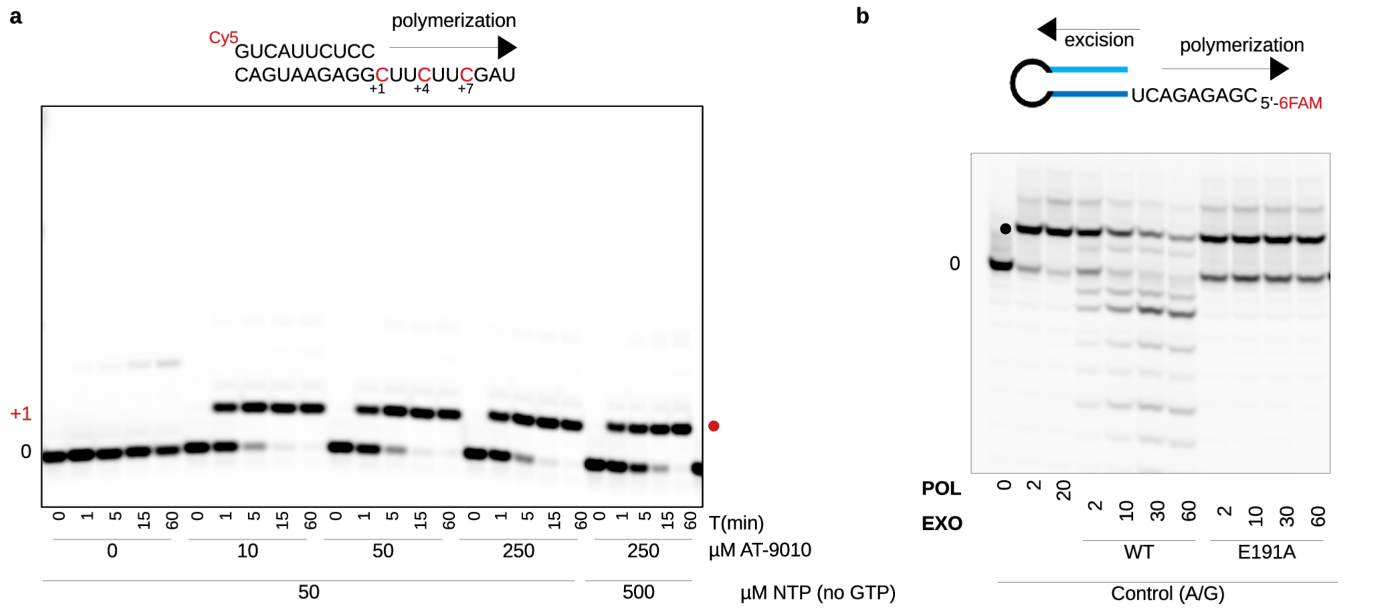

Supplementary Fig. 3. Incorporation of AT-9010 by the SARS-CoV-2 RTC, and excision by the nsp14/10 exonuclease complex (ExoN). (a) Timecourse of incorporation of AT-9010 at the first position (red dot) in the presence of ATP, UTP and CTP (no GTP) at indicated concentrations, showing RNA chain termination. Reactions were run in duplicate at multiple NTP concentrations for both SARS-CoV and SARS-CoV-2 with comparable results (b) RNA extension (POL) with ATP + GTP (+2 product, black dot) followed by excision (EXO) with ExoN wild-type (WT) and active-site mutant (E191A). Reactions were run in duplicate, and additionally following AT-9010 and STP incorporation, confirming no excision of incorporated products by the E191A ExoN active-site mutant. The RNA marked at position 0 on each gel corresponds to the size of the fluorescently labeled primer shown above each gel, prior to elongation. Source data are provided as a Source Data file.

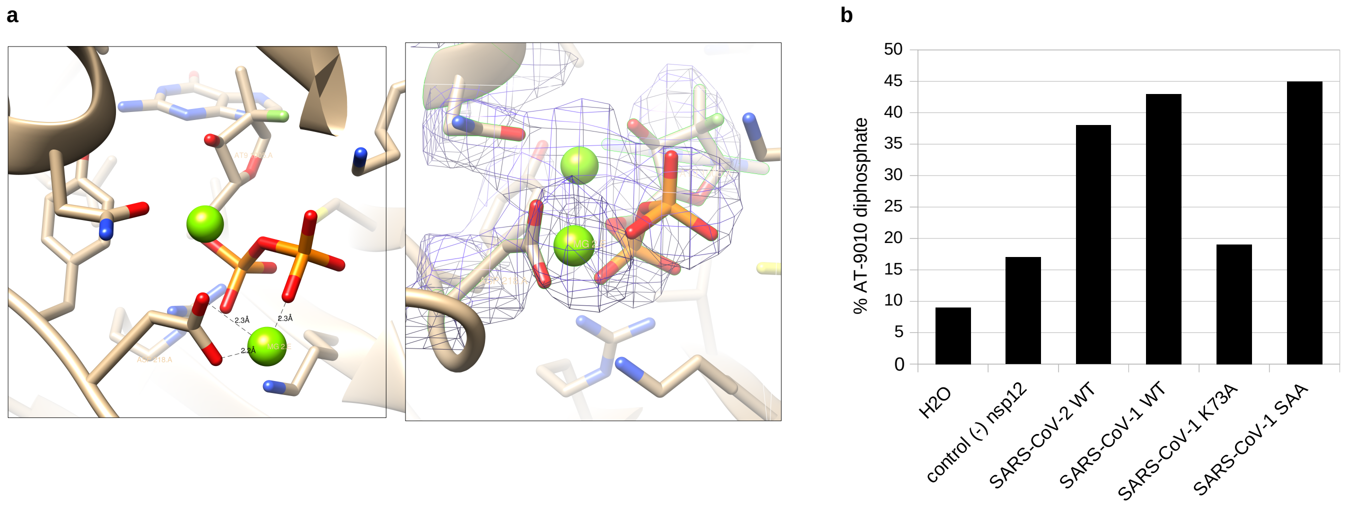

Supplementary Fig. 4. Density and coordination distances of metal ions with the AT-9010-DP, and hydrolysis of the γ-phosphate in the NiRAN active-site. (a) left; distances in Å between the additional magnesium ion, one non-bridging oxygen of the AT-9010-DP β-phosphate, and carboxyl group of D218 of the NiRAN domain. Right; associated cryoEM map represented at 3.5σ representative of the general map of the entire complex at 2.98 Å resolution. (b) Percentage of AT-9010 diphosphate formed following incubation for 1 hour at 37 °C with either no enzyme (control), nsp12 WT enzymes, or NiRAN (K73A) or RdRp (SAA) active-site mutants. Samples were heat-inactivated at 70 °C to promote dissociation of bound AT-9010 and purified through microspin columns, prior to separation on HPLC C18 columns for analysis. Source data are provided as a Source Data file.

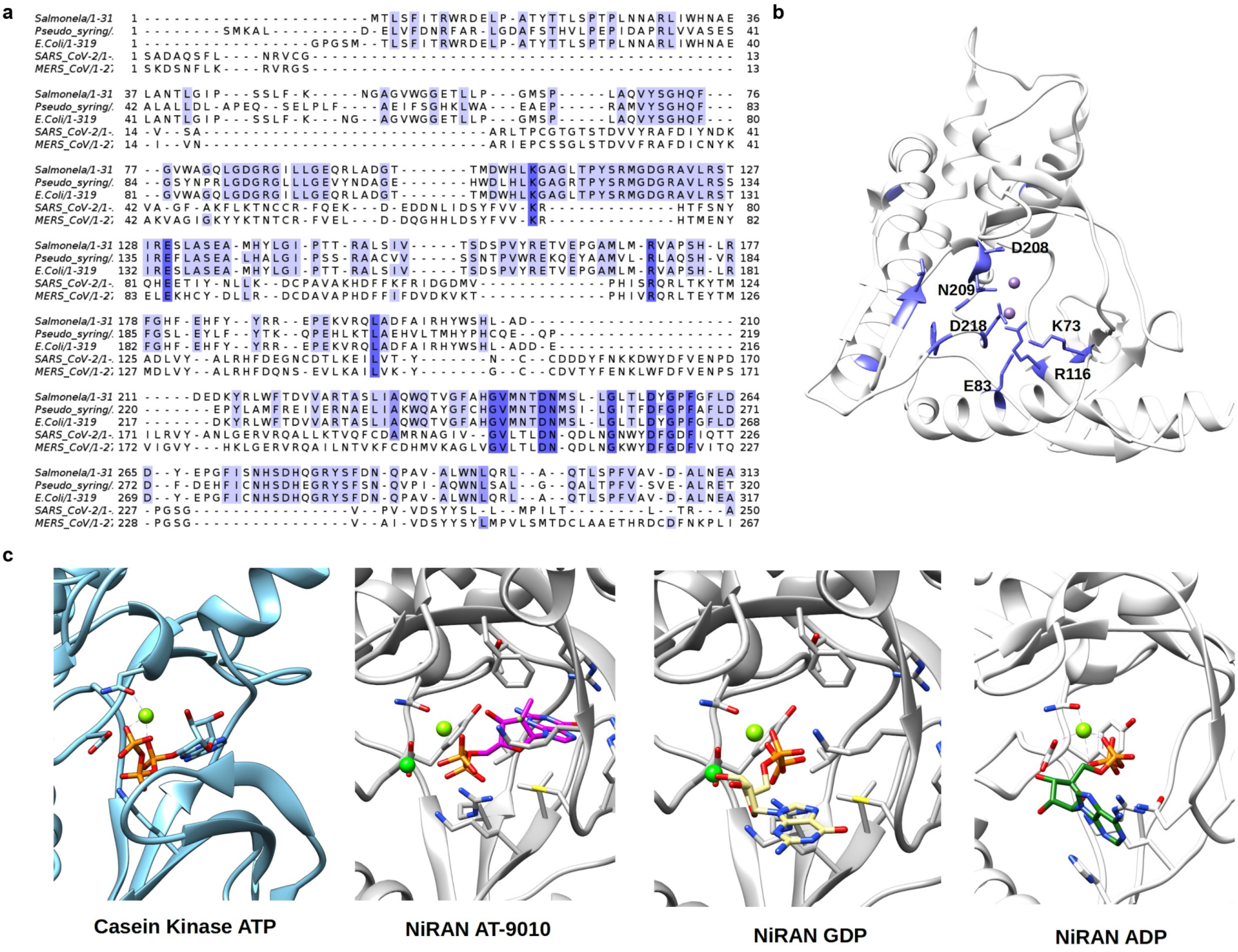

Supplementary Fig. 5. Pseudo-kinase fold and conservation of the NiRAN domain of nsp12. (a) Sequence alignment derived from structural superimposition of several pseudo-kinase structures and the NiRAN domain. (b) Sequence conservation (in blue) plotted onto the NiRAN domain structure. For A and B conserved residues are in deep blue. (c) Nucleotide ligand binding into the Casein kinase (PDB: 1CSN, doi:10.2210/pdb1CSN/pdb) and comparative analysis of the NiRAN catalytic site with AT-9010-DP, GDP (PDB: 7CYQ, doi:10.2210/pdb7CYQ/pdb), and ATP from the SelO structure (PDB: 6EAC, doi:10.2210/pdb6eac/pdb), showing the different nucleotide binding orientations.

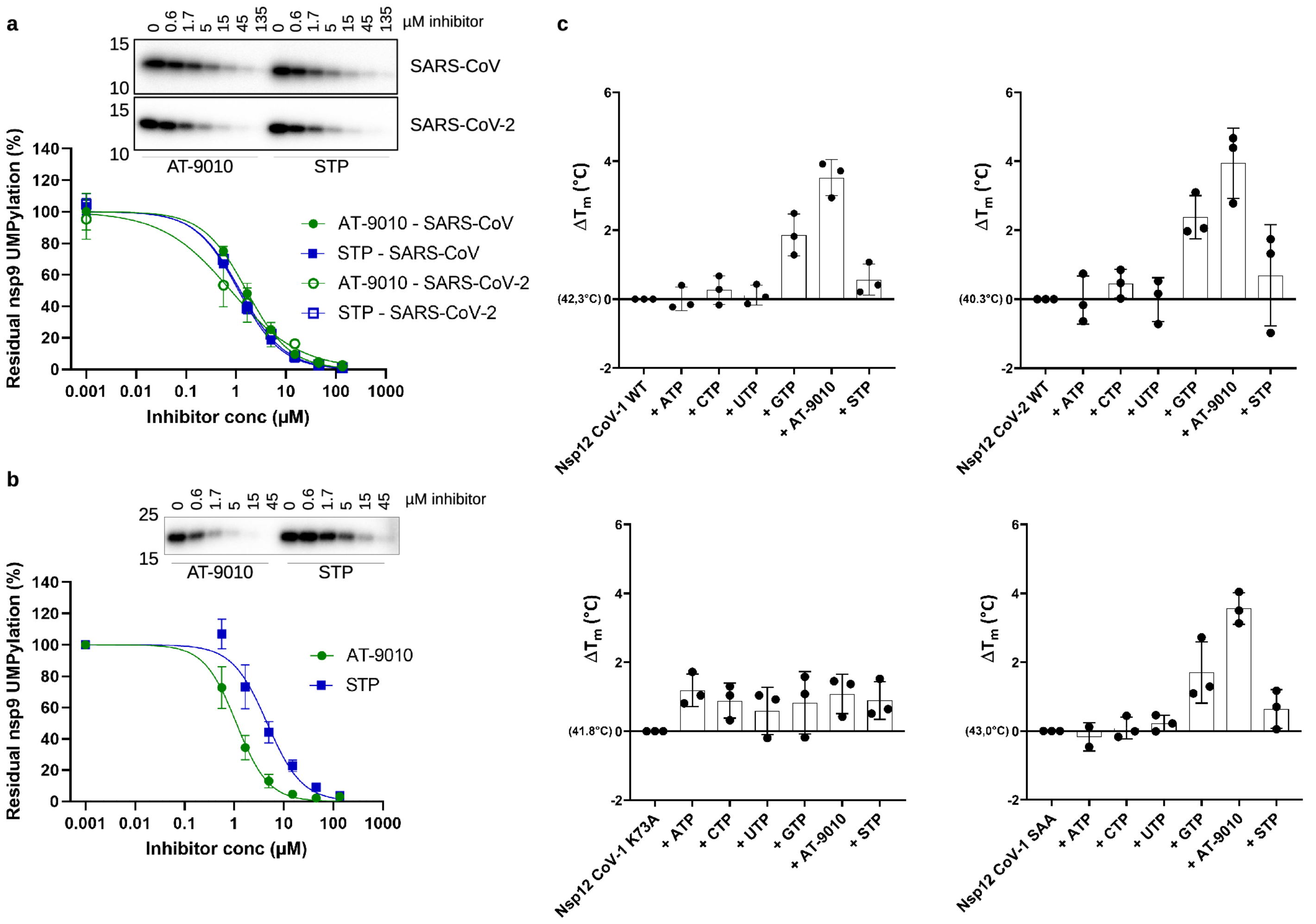

Supplementary Fig. 6. Inhibition of NiRAN nucleotide transferase activity and NA binding to SARS-CoV and SARS-CoV-2 nsp12. (a) Labelling of nsp9 by nsp12 in the presence increasing concentrations of AT-9010 (green) or STP (blue), and a constant concentration of α^{32}P-UTP. Molecular weight sizes shown on left of gel are based on coomassie staining of the same gel, as shown in Source Data. Quantitation of radiolabelled (inset) is plotted as a function of inhibitor concentration (graph). Total intensity of labelling was quantified with ImageQuant software, and plotted at % residual activity. Data are presented as mean values +/- SEM (n=4). (b) Same as (a) using nsp8 instead of nsp9 for SARS-CoV only. Data are presented as mean values +/- SEM (n=3). (c) Thermal shift analysis of nsp12 in the presence of NTP or NAs as indicated. Nsp12 is either WT, NiRAN mutant K73A or RdRp mutant SAA as indicated. Reactions were run in triplicate, Data are presented as mean values +/- SD. Source data are provided as a Source Data file.
