# Supplementary material for: A dual mechanism of action of AT-527 against SARS-CoV-2 polymerase
Source: Nat Commun. 2022 Feb 2;13:621. doi: 10.1038/s41467-022-28113-1 (PMC8810794; doi:10.1038/s41467-022-28113-1)
Supplement: Supplementary file 3 — Reporting Summary [file 41467_2022_28113_MOESM3_ESM.pdf]

nature research

Corresponding author(s): Bruno Canard
Last updated by author(s): Shannon 2021/12/16

Reporting Summary

Nature Research wishes to improve the reproducibility of the work that we publish. This form provides structure for consistency and transparency in reporting. For further information on Nature Research policies, see our Editorial Policies and the Editorial Policy Checklist.

Statistics

For all statistical analyses, confirm that the following items are present in the figure legend, table legend, main text, or Methods section.

n/a	Confirmed	
☐	☒	The exact sample size (*n*) for each experimental group/condition, given as a discrete number and unit of measurement
☐	☒	A statement on whether measurements were taken from distinct samples or whether the same sample was measured repeatedly
☒	☐	The statistical test(s) used AND whether they are one- or two-sided<br>*Only common tests should be described solely by name; describe more complex techniques in the Methods section.*
☒	☐	A description of all covariates tested
☒	☐	A description of any assumptions or corrections, such as tests of normality and adjustment for multiple comparisons
☐	☒	A full description of the statistical parameters including central tendency (e.g. means) or other basic estimates (e.g. regression coefficient) AND variation (e.g. standard deviation) or associated estimates of uncertainty (e.g. confidence intervals)
☒	☐	For null hypothesis testing, the test statistic (e.g. F, t, r) with confidence intervals, effect sizes, degrees of freedom and P value noted<br>*Give P values as exact values whenever suitable.*
☒	☐	For Bayesian analysis, information on the choice of priors and Markov chain Monte Carlo settings
☒	☐	For hierarchical and complex designs, identification of the appropriate level for tests and full reporting of outcomes
☒	☐	Estimates of effect sizes (e.g. Cohen's d, Pearson's r), indicating how they were calculated

Our web collection on statistics for biologists contains articles on many of the points above.

Software and code

Policy information about availability of computer code

Data collection	SerialEM 3.7
Data analysis	Relion-3.1.0, cryoSPARC v2.15.0, Phenix -1.19_4085, Graphpad Prism version number 9.3, UCSF Chimera version number 1.15, Image Quant TL version 8.1.0.0

For manuscripts utilizing custom algorithms or software that are central to the research but not yet described in published literature, software must be made available to editors and reviewers. We strongly encourage code deposition in a community repository (e.g. GitHub). See the Nature Research guidelines for submitting code & software for further information.

Data

Policy information about availability of data

All manuscripts must include a data availability statement. This statement should provide the following information, where applicable:

- Accession codes, unique identifiers, or web links for publicly available datasets
- A list of figures that have associated raw data
- A description of any restrictions on data availability

The coordinates and structure factors for SARS-CoV-2 nsp7:nsp8:nsp12:RNA:AT-9010 quaternary structure have been deposited with the following accession codes: in the PDB : 7ED5, and in the EMDB: EM-31061 respectively. The biochemical incorporation, excision and NiRAN inhibiton data generated in this study are provided in the Supplementary Information and Source Data file. Data processing and interpretation for the biochemical data is also available in the Source data file.

Field-specific reporting

Please select the one below that is the best fit for your research. If you are not sure, read the appropriate sections before making your selection.

☒ Life sciences ☐ Behavioural & social sciences ☐ Ecological, evolutionary & environmental sciences

For a reference copy of the document with all sections, see nature.com/documents/nr-reporting-summary-flat.pdf

Life sciences study design

All studies must disclose on these points even when the disclosure is negative.

Sample size	Biochemistry experiments were done with three or more repeats across a range of nucleotide and/or analogue compound concentrations. For exonuclease assays, two different RNAs were tested to ensure that results were not impacted by RNA sequence or secondary structure. For all biochemistry experiments, both SARS-CoV and SARS-CoV-2 polymerase complexes were tested, and all experiments were run by at least two different persons. Results were reproducible and highly consistant across all data sets.
Data exclusions	Two random outliers were excluded from NiRAN nsp9-UMPylation inhibition analysis due to obvious errors in sample preparation (I.e intensity values ~20X higher than predicted based on other replicated). Values shown in source data file.
Replication	at least three independent experiments were run for each experiment, with 3 replicates per experiment. Each experiment was additionally run with at least 2 different RNAs to control for variability. All experiments yielded consistant results in line with the overall conclusions.
Randomization	For the most part, randomisation was not relavent to our study. For biochemical studies, sample order was varied to aid in reducing potential experimental error and sample loading order on gels was varied, to aid in reducing sample bias due to sample bleeding into neighbouring wells.
Blinding	Blinding was not relevant to this study. All results were quantifiable and therefore unable to be manipulated. Additionally, experimental repeats were regularly analysed by two independent researchers.

Reporting for specific materials, systems and methods

We require information from authors about some types of materials, experimental systems and methods used in many studies. Here, indicate whether each material, system or method listed is relevant to your study. If you are not sure if a list item applies to your research, read the appropriate section before selecting a response.

Materials & experimental systems

n/a	Involved in the study	
☒	☐	Antibodies
☐	☒	Eukaryotic cell lines
☒	☐	Palaeontology and archaeology
☒	☐	Animals and other organisms
☒	☐	Human research participants
☒	☐	Clinical data
☒	☐	Dual use research of concern

Methods

n/a	Involved in the study	
☒	☐	ChIP-seq
☒	☐	Flow cytometry
☒	☐	MRI-based neuroimaging

Eukaryotic cell lines

Policy information about cell lines

Cell line source(s)	sf21
Authentication	ThermoFisher Product No.<br>11497013<br>Lot No.<br>2045665<br>Date of Manufacture<br>28Dec2018
Mycoplasma contamination	Negative
Commonly misidentified lines (See ICLAC register)	not concerned as per ICLAC doc. No commonly misidentified lines according to ICLAC register.
