# Supplementary material for: A dual mechanism of action of AT-527 against SARS-CoV-2 polymerase
Source: Nat Commun. 2022 Feb 2;13:621. doi: 10.1038/s41467-022-28113-1 (PMC8810794; doi:10.1038/s41467-022-28113-1)
Supplement: Supplementary file 4 — Supplementary Table 1 [file 41467_2022_28113_MOESM4_ESM.pdf]

File Name: Supplementary Movie 1
Description:
SARS-CoV-2 replication complex with RNA in elongation mode stalled by AT-9010 5'MP chain-terminated RNA (referred to as (+1) in the text), and a second AT-9010 bound in the NTP binding site (referred to as (-1) in the text).

File Name: Supplementary Movie 2
Description:
SARS-CoV-2 replication complex focus on nsp12-NiRAN domain bound to AT-9010-5'DP.
